# The Role of Contrast-Enhanced Ultrasound in Selection Indication and Improveing Diagnosis for Transthoracic Biopsy in Peripheral Pulmonary and Mediastinal Lesions

**DOI:** 10.1155/2015/231782

**Published:** 2015-05-18

**Authors:** Song Wang, Wei Yang, Hui Zhang, Qian Xu, Kun Yan

**Affiliations:** ^1^Key Laboratory of Carcinogensis and Translational Research of Ministry of Education, Department of Ultrasound, Peking University Cancer Hospital & Institute, Beijing 100142, China; ^2^Department of Ultrasound, Harbin First Hospital, Harbin, Heilongjiang 150010, China

## Abstract

*Objective*. To investigate the value of contrast-enhanced ultrasound (CEUS) in transthoracic biopsy of peripheral lung and mediastinal lesions. *Methods*. Of 142 patients, 82 patients received CEUS before biopsy and were defined as CEUS group. The remaining 60 patients only underwent conventional ultrasound (US) before biopsy and were served as US group. The information of CEUS was used for selecting indication and instructing biopsy. The imaging features, number of punctures, diagnostic successful rate, and complication rate between the two groups were compared. *Results*. Necrosis was demonstrated in 43.9% of the lesions in CEUS group and in 6.7% of US group (*P* < 0.001). Detection rate of lesion hidden in pulmonary atelectasis in CEUS group was 13.4%, which was statistically higher than 1.7% of US group (*P* = 0.013). The diagnostic success rate was 96.3% for CEUS group and 80% for US group, respectively (*P* = 0.002). The average number of punctures was 2.5 ± 0.7 and 2.6 ± 0.6, respectively. There was no significant difference in complications between CEUS group and US group. *Conclusions*. CEUS could play an important role in selecting proper indication and improving diagnostic accuracy rate of lung biopsy.

## 1. Introduction

Lung cancer is one of the most threatening tumors in the world, the morbidity and mortality of which are growing rapidly in the past decades. Until now, there are still a significant number of patients who died from lung cancer [1]. The ultrasound-guided biopsy of peripheral lung lesions which is convenient, safe, and repeatable gradually drawn great attentions [2–4]. The accuracy of ultrasound-guided biopsy is comparable to CT-guided biopsy [5]. However, it is still difficult to find out more detailed information about tissue structure from gray-scale and color Doppler ultrasound, such as whether there is lesions hidden in atelectasis lungs, whether there is necrosis within lesions or the area of necrotic lesions. All these factors mentioned above may cause the possibility of inaccurate biopsy or false negative results [6]. For the past few years, SonoVue, which is the representative of a new generation of ultrasound contrast agents, can characterize the microvascular perfusion within lesions and can be gradually applied to the diagnosis of peripheral lung lesions. Cao et al. [7] suggested that CEUS contributed to identifying tumor tissues and necrotic tissues and to increasing diagnostic rate of biopsy. In this study, we performed a CEUS examination before each patient potential to receive biopsy in order to select the proper indication. Then we expected to further direct the puncture by CEUS and to improve diagnostic accuracy rate.

## 2. Materials and Methods

### 2.1. Subjects and Methods

This study was approved by our local ethics committee, and written informed consent was obtained from each patient before the CEUS examination and biopsy procedures.

### 2.2. Patient Population

From July 2011 to July 2013, 150 patients who were diagnosed as peripheral lung or mediastinal lesions by chest CT and were recommended for ultrasound-guided transthoracic biopsy in our department were enrolled in this study. Among them, 95 were males and 55 females and their age ranged from 21 to 80 years (median, 59 years old). Of the 150 patients, 60 who underwent biopsy after conventional US examination (before June 2012) constituted the US group, because the CEUS was not performed for lung lesion until June 2012 in our department. The other 90 patients underwent CEUS before biopsy during June 2012 to July 2013. Among the 90 patients, 8 did not receive biopsy after CEUS because of being not suitable for the procedure. The remaining 82 patients constituted of CEUS group. All the patients' ages, male to female ratios, lesion locations, and malignant to benign ratios were collected and there were no statistically significant differences between the US and CEUS groups. The baseline characteristics of the patients were shown in Table 1.

### 2.3. Contrast Agent and Sonography Procedures

For the CEUS group, patients underwent CEUS before biopsy. The sonography contrast agent used was SonoVue (Bracco, Italy), supplied as a lyophilized powder and reconstituted with 5 mL of saline to form a homogeneous microbubble suspension that contains 8 *μ*L/mL of sulfur hexafluoride stabilized by a phospholipid shell. The mean microbubble diameter is 2.5 *μ*m with a pH value of 4.5~7.5. SonoVue was administered IV as 2.4 mL boluses through the antecubital vein in 2~3 seconds. CEUS was performed using the Logiq E9 sonography system (GE,USA) with real-time gray-scale contrast tuned imaging and a 3.0~5.0 MHz C5-1 probe (GE,USA).

With the reference of chest CT, conventional US was performed to investigate the lesions' size, location, and sonographic features. Contrast-enhanced imaging was then initiated, and the acoustic power output was adjusted to about 0.11 to 0.13 mechanical index on the basis of the lesion depth and body habitus of the patient. On contrast, agent administration, the perfusion and enhancement patterns, time to enhancement, and necrosis (anechoic) area of the target lesions were continuously observed over 5 minutes. The dynamic image was recorded on the hard disk of the sonography machine. CEUS analysis was performed on the basis of review of stored sonographic clips.

Finally, the biopsy puncture plan was established by collecting the information of the lesions' location, size, necrosis area, pulmonary atelectasis, and pleural effusion.

### 2.4. Biopsy Indication and Method

The indication for ultrasound guided transthoracic biopsy of peripheral pulmonary lesions was as follows: (1) accessibility of lesions via a percutaneous approach with ultrasound; (2) lesion larger than 1.5 cm in diameter and larger than 2 cm in deep location; (3) lesion has at least 1.5 cm thickness of solid content; (4) patients can well control breath; (5) international normalized ratio was no greater than 1.6 and platelet count was greater than 80000/L.

For the biopsy procedures in this study, sonography system (Alpha 10, Aloka, Japan, and Logiq E9, GE, USA) with 3.5~5.0 MHz small-sector convex probes accessorized for biopsy was used for biopsy. Color Doppler imaging was routinely used to delineate large vessels and abnormal arteries so the operator could avoid puncturing them during biopsy. All patients fasted for at least 8 hours before the procedure. In the CEUS group, CEUS was performed 30~60 minutes before the biopsy, and the information was used for planning the biopsy route and sampling site.

Biopsy was performed using an 18-gauge automatic cutting needle (Bard Magnum, Bard, USA). After careful planning, the skin was sterilized, and local anesthesia was applied using 1% lidocaine (Liduokayin, Yimin). After the probe was fixed and the guiding needle was inserted into the chest wall, the biopsy needle was inserted for biopsy. Patients were told to hold their breath when the needle was advanced into or was withdrawn from the lung lesion. It should be noted that the biopsy route should avoid penetrating aerated lung or large vessels. The number of puncture attempts was decided by the quantity and color of the specimen obtained. The whole biopsy procedure was continuously monitored using conventional US. The specimens obtained during the biopsy were fixed in 10% formalin.

After biopsy, the puncture site was routinely checked for active bleeding or other complications. The patient stayed in the hospital for at least 1 hour after the procedure and was told to continue fasting for 4 hours after biopsy. The specimens were sent to the pathology department for histologic and cytologic examinations by two experienced pathologists.

### 2.5. Imaging Diagnosis

Information such as lesion size, location, necrosis, and pulmonary atelectasis was recorded and the difference between CEUS group and US group was compared. Diagnosis of necrosis in CEUS group was determined if CEUS showed the area in the lesion was complete absence of enhancement during arterial phase and parenchymal phase [8]. Diagnosis of necrosis in US group was determined if conventional US showed that there was anechoic area within lesions and clear boundaries, and CDFI showed no blood flow within the anechoic area. Diagnosis of pulmonary atelectasis in CEUS group was determined if CEUS showed early and homogeneous enhancement and slow wash out during parenchymal phase. The straight or branch-like tumor vessels were displayed in the enhancement area [9]. Diagnosis of pulmonary atelectasis in US group: conventional US showed the lesion was wedge-shape and iso-echoic, the tip of lesion pointed to pulmonary hilus. Scattered air or liquid bronchogram toward the peripheral parenchyma was visible, and regular blood flow was demonstrated by CDFI [10, 11]. According to contrast enhanced CT, the detection rate for necrosis and pulmonary atelectasis was decided in the two groups.

### 2.6. Final Diagnosis

**Figure 1:**
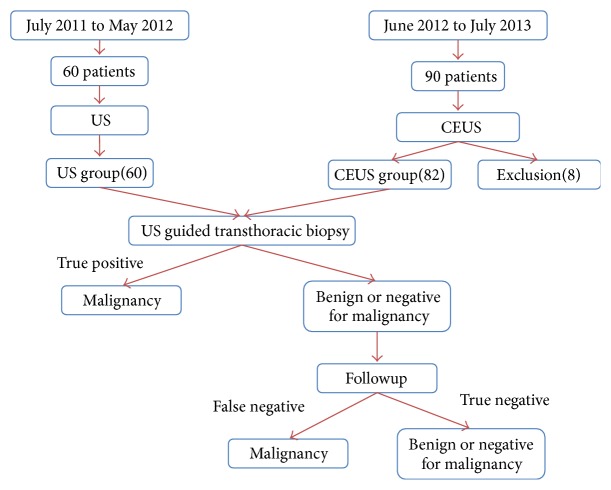
Flow chart shows algorithm for diagnosis of lung lesion.

The final diagnosis was made as definite diagnosis, descriptive diagnosis, and unable diagnosis based on comprehensive diagnosis of all pathological specimens from two experienced pathologists. Definite diagnosis was considered correct if the pathologic diagnosis confirmed malignancy. Benign diagnosis and descriptive diagnosis need to be confirmed with other imaging examinations and one-year followup. If the other imaging examination and followup confirm the diagnosis, the diagnosis was considered correct. It was considered a failure if no tissues or undeterminable necrotic tissues were described in the descriptive diagnosis (Figure 1).

### 2.7. Statistical Analysis

All analyses were performed using SPSS 21.0 statistical analysis software (IBM, USA). Enumeration data were given as mean ± SD and were analyzed with an unpaired *t* test. Categorical variables were analyzed with Pearson *χ* ^2^ and Fisher exact tests. *P* < 0.05 was considered statistically significant.

## 3. Results

### 3.1. Biopsy Indication Selection with CEUS

**Figure 2:**
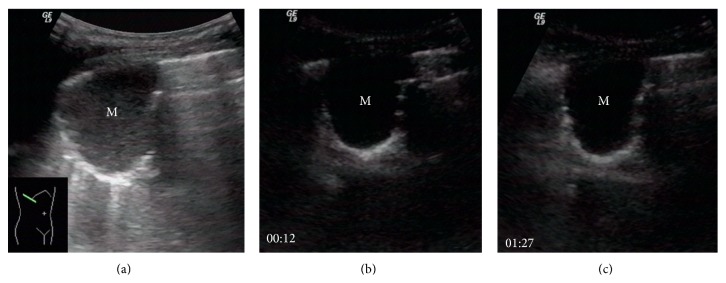
Images from a 61-year-old female with a cough and chest pain for 2 months. (a) B-mode sonogram showed a 3.8 × 2.9 cm lesion (M) with hypoecho texture in inferior lobe of right lung. (b) CEUS obtained 12 seconds after administration of SonoVue showed a lesion with complete absence of enhancement (M). (c) CEUS obtained 1 minute and 27 seconds after injection of SonoVue showed that the lesion still had absence of enhancement (M). Biopsy was cancelled because no neoplasm was revealed on CEUS. CT scan conformed that the whole lesion represented as necrosis tissue.

Based on CEUS finding, eight patients were considered nonsuitable for ultrasound guided transthoracic biopsy, including the fact that three cases had the lesions bigger than 80% of the necrotic (Figure 2), two patients had small tumors (the diameter was less than 2 cm) located deeply behind the pulmonary atelectasis, and three patients were diagnosed as consolidation or atelectasis lungs. The results were summarized in Table 2. The diagnosis for these excluded cases was obtained by other methods (surgery, CT guided puncture, or follow-up visit for 12 months). Of them, three cases were lung consolidation, two cases were small cell carcinoma, two cases were adenocarcinoma, and one case was squamous carcinoma. Finally, the remaining 82 cases who received CEUS and then biopsy were regarded as CEUS group.

### 3.2. The Final Diagnosis of the Two Groups

CEUS group consisted of 82 cases. There were 61 cases malignancies, including 23 cases of squamous carcinoma, 25 cases of adenocarcinoma, 6 cases of small cell carcinoma, 5 cases of metastatic carcinoma, and 2 cases of neuroendocrine carcinoma. There were 21 cases of benign tumors, including 6 cases of inflammatory pseudotumors, 7 cases of pneumonia consolidation, 3 cases of tuberculosis, 2 cases of lymphoma, and 3 cases of thymoma.

In US group, there were 36 cases of malignancies, including 16 cases of squamous carcinoma, 13 cases of adenocarcinoma, 3 cases of small cell carcinoma, 2 cases of metastatic carcinoma, and 2 cases of neuroendocrine carcinoma. There were 24 cases of benign tumors, including 8 cases of inflammatory pseudotumors, 6 cases of pneumonia consolidations, 3 cases of tuberculosis, 4 cases of lymphomas, and 3 cases of thymomas. The results were summarized in Table 3.

### 3.3. Comparison of Imaging Features between US Group and CEUS Group

**Figure 3:**
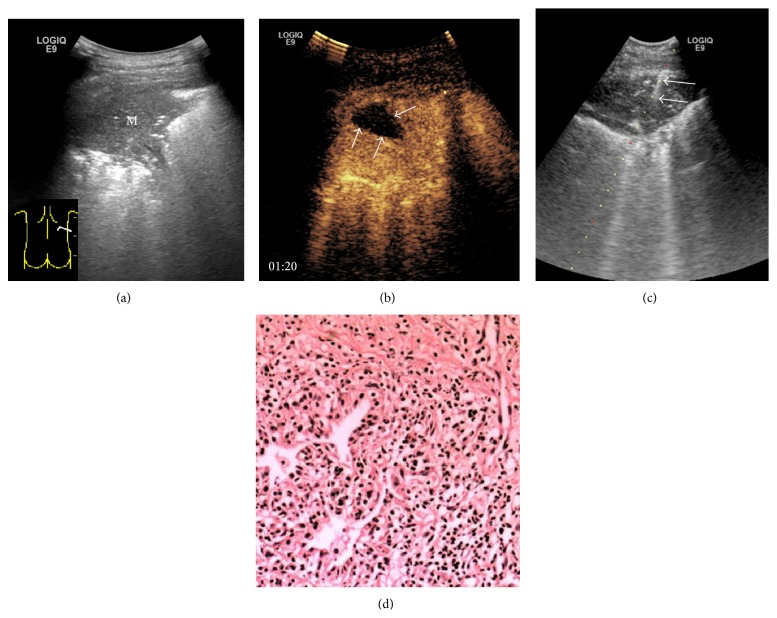
Images from a 68-year-old male with chest pain for a week. (a) B-mode sonogram showed a heterogeneous lesion (M) with air-bronchogram in inferior lobe of right lung. (b) CEUS showed a regular necrosis (↑) in the center of lesion. (c) US-guided transthoracic biopsy was performed after CEUS and biopsy was targeted in the enhanced area (↑) to avoid necrotic area. (d) Pathological result from biopsy demonstrated a benign pulmonary inflammatory pseudotumor (hematoxylin-eosin, original magnification ×200).

**Figure 4:**
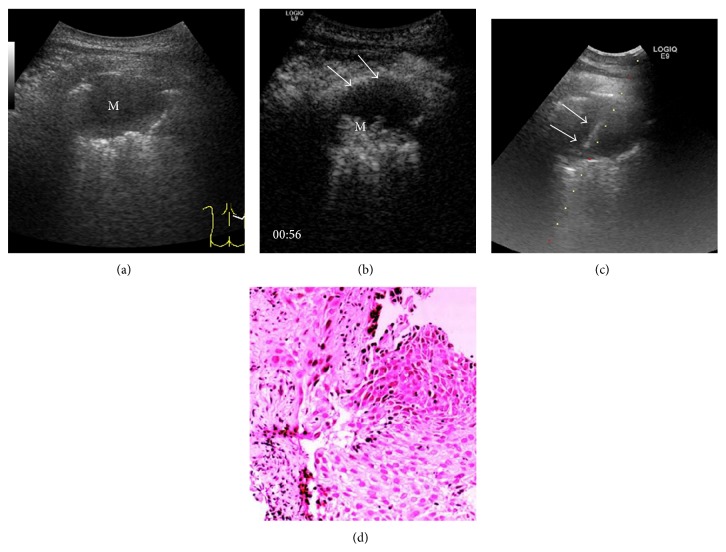
Images from a 71-year-old male with a history of chest pain and hemoptysis. (a) B-mode sonogram showed a hypoecho lesion (M) in middle lobe of right lung. (b) CEUS obtained 56 seconds after injection of SonoVue showed an irregular necrosis in the majority of lesions (↑) and only small area (M) had slight enhancement in the border. (c) US-guided transthoracic biopsy punctured through the necrosis area and targeted the enhanced area (↑). (d) Pathological result from biopsy demonstrated a poorly differentiated pulmonary adenocarcinoma with necrosis (hematoxylin-eosin, original magnification ×200).

The size of tumors was 6.5 ± 2.8 cm and 6.7 ± 3.4 cm in CEUS group and US group and had no statistical difference between these two groups. The minimum size of tumors was 2.4 cm in CEUS group and 2.3 cm in US group. The detectable rate of necrosis in CEUS group was 43.9%, which was statistically higher than that of US group (6.7%, *P* < 0.001). In CEUS group, necrosis with regular shape was found in 5 cases of benign lesions (Figure 3), and necrosis with irregular shape was found in 31 cases of malignant lesions (Figure 4). In malignant lesions, the increased size of lesions was accompanied by higher rate of necrosis. The necrotic detection rate of CEUS group was 30% for lesions less than 3 cm, 37.5% for lesions between 3 cm and 5 cm, and 50% for lesions bigger than 5 cm. The detection rate of lesion hidden in pulmonary atelectasis was 13.4% in the CEUS group, which was statistically higher than the US group (1.7%, *P* = 0.013) as shown in Table 4.

### 3.4. Comparison of Diagnostic Accuracy between CEUS Group and US Group

**Figure 5:**
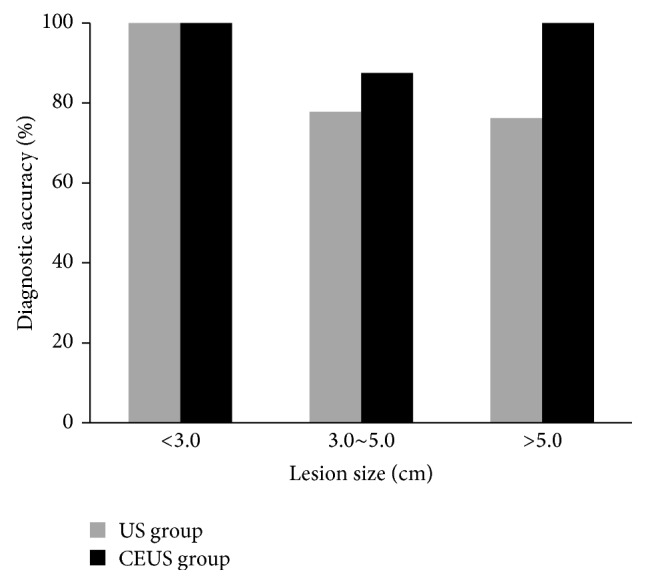
Bar graph shows diagnostic accurate rate for CEUS group (black) and US group (light gray) in lung lesions with different size. There was statistically significant difference between CEUS group and US group in diagnostic accurate rate of lung lesions >5.0 cm (100% (*n* = 48/48) versus 76.2% (*n* = 32/42), *P* = 0.002). The diagnostic accurate rate of lung lesions 3.0~5.0 cm in CEUS group was higher than that in US group but had no significant difference (87.5% (*n* = 21/24) versus 77.8% (*n* = 7/9), *P* = 0.488). CEUS, contrast enhanced ultrasound; US, ultrasound.

Based on the final diagnosis, the diagnostic accurate rate was 96.3% in CEUS group, which was statistically higher than 80% of US group (*P* = 0.002). There was no statistical difference in the diagnostic accurate rate of lesions less than 3 cm between the two groups. The diagnostic accurate rate of lesions between 3~5 cm was slightly higher in CEUS group than that in US group, but there was no statistical difference. However, the diagnostic accurate rate of lesions bigger 5 cm was statistically higher in CEUS group than that in US group (*P* < 0.001). This information was shown in Table 4 and Figure 5.

### 3.5. Analysis of False-Negative Diagnosis in Conventional Ultrasonography Group

We performed a post hoc analysis on a subgroup of 12 patients in US group. These patients showed a negative diagnosis for malignancy from the initial biopsy but malignancy could not be excluded due to clinical data or enhanced CT. Repeat biopsy was performed in these patients to confirm the diagnosis. All of the 12 lesions were diagnosed as malignant at the second biopsy. Result of pathological diagnosis for initial puncture was shown in Table 5.

### 3.6. Complication Rate and Puncture Attempts of Two Groups

The average number of punctures was 2.5 ± 0.7 in CEUS group and 2.6 ± 0.6 in US group, which meant that there was no significant difference between these two groups (*P* = 0.462). No serious complication or procedure related death occurred in these two groups. One case had asymptomatic aerothorax and one case had hemoptysis. The complication rate was 2.4% in CEUS group and 3.3% in US group, which indicated that there was no statistical difference between these two groups (*P* = 1.000), as shown in Table 6.

## 4. Discussion

Recently, more attentions were paid to ultrasonography technology because of the following advantages: real-time, convenience, flexibility, repeatability, and nonradiation. Considering the development of ultrasound guided transthoracic puncture technology, higher accuracy of pathologic sampling and less severe complication are achieved [12]. However, it is difficult to identify benign or malignancy or whether there is necrotic tissue within lesions by gray-scale and color Doppler ultrasound [13, 14]. Ultrasonic contrast agent is blood pod agent, which will not leak out of vessels. It is easier to display the microvascular within lesions using contrast-enhanced agent compared to CT scan [15–17].

Cao et al. [7] reported the initial result that puncture diagnostic success rate was elevated by CEUS examination. CEUS could show the necrotic area within lesions and guide puncture biopsy to avoid necrotic area [18]. Our study proposed that CEUS could not only sensitively showed the inner structure of lesions, but also help to screen the biopsy indication, then reduce unnecessary damage to patients. After CEUS examination, necrosis more than 80% of lesions was found in 3 cases. 2 lesions less than 2 cm were found deeply in the atelectasis lung tissue. Three cases were diagnosed as consolidated lung tissue by CEUS. These patients were excluded from invasive biopsy and all of them got final diagnosis by other methods.

Görg et al. [19, 20] demonstrated that the blood supply of lung lesion came from pulmonary artery if the enhancement appeared before 10 s after administration of SonoVue. On the other hand, if the enhancement appeared later than 10 s after contrast agent injection, the blood supply of lung lesions was indicated as bronchial arteries. These parameters could be used to identify consolidated lung tissues and malignant lesions. Di Vece et al. [21] considered that the enhancement phase of primary lung cancer begun from bronchial arterial phase. The strength of enhancement was lower than atelectasis lungs, which was meaningful for the diagnosis of tumor located in the atelectasis lungs. Consistent with the findings of di Vece, the present study demonstrated that CEUS assessment prior to biopsy increased the detectable rate of lesions hidden in atelectasis lungs compared to US group (13.4% versus 1.7%, *P* = 0.013). Compared to conventional ultrasound, CEUS assessment prior to biopsy facilitated the detection of pathologic characteristics of lesions, which provided important information for selecting indication and improving the success rate of biopsy.

It is known that the failure of puncture is mainly due to the little amount of sample tissues or most of the sample tissues are necrotic [22]. Since CEUS could detect the necrotic tissues in the lesions, CEUS would improve the pathologic diagnostic accuracy. In this study, the detectable rate of necrosis in CEUS group was significantly higher than in US group (43.9% versus 6.7%). Furthermore, the detectable rate of necrosis in CEUS group was 30% for lesions less than 3 cm, 37.5% for lesions between 3 cm~5 cm, and 50% for lesions bigger than 5 cm. Necrotic tissues were easily found in the malignant lesions and bigger tumors, which was consistent with the report of Cao et al. [7]. This may be related to the blood supply of tumors: the growth of vasculatures could not meet the need of tumor for fast growth, and then necrosis tissues occurred. Meanwhile, the inner necrotic area of malignant lesions appeared to have irregular shape or uneven distribution in 31 cases of the CEUS group. However, the necrotic area of benign lesions appeared to have regular shape such as quasicircular or oval shape with clear necrotic boundaries. Therefore, CEUS should be applied to further assess the necrotic tissues within lesions if CT, gray-scale ultrasound, or X-ray examination indicated that there might be malignant lesions.

Similar to the blood supply of liver, there are two systems in the lungs: the pulmonary arteries and bronchial arteries. The success rate and accuracy of puncture could significantly be improved if the area with enhancement at bronchial arteries is selected as the site of puncture. Pan et al. [23] reported that the successful rate was related to the size of lesions. Our study further demonstrated that, for the lesions less than 3 cm, conventional ultrasound could achieve similar diagnostic accuracy because of the low rate of necrosis. For the bigger lesions (3–5 cm in diameter), although there was no statistical difference, the CEUS assessment helped to display whether there was necrotic tissue or consolidated lung tissues within lesions, which enhanced the confidence of doctors. For lesions bigger than 5 cm, CEUS assessment significantly increased the diagnostic rate. Therefore, this study demonstrated that the assessment of CEUS before biopsy was recommended if the size of pulmonary lesion was bigger than 3 cm.

There were still some limitations in this study. First, the two groups classified in this study were not randomized. We divided the two groups in June 2012 as time point, because CEUS was routinely used in peripheral lung lesions before biopsy after this time. With the experience collected, the biopsy success rate in later years tended to increase. Second, either CEUS or US had difficulty to show deeply located lung lesions through normal lung tissue. For the patients with central type of lung tumor and without enough ultrasound windows, CT scan was necessary to provide diagnostic information. Third, although CEUS facilitated the accuracy of puncture compared to conventional ultrasound, there was high risk to guide the needle biopsy to deeply located lesions. Cautions should be paid to CEUS guided puncture for the lesions deeply located in the atelectasis lungs. Last, in this study, the enhancement pattern for benign or malignant lesions in CEUS group was overlapped in some cases; thus CT scan should double check the benign lesions diagnosed by CEUS and follow-up visit is necessary to avoid misdiagnosis.

## 5. Conclusions

The CEUS assessment prior to biopsy easily detects the lesions hidden within lung tissues and the necrotic lesions, which provides useful information to screen puncture indication and guide the biopsy in the right way. Compared to the conventional ultrasound, CEUS assessment facilitates the success rate of puncture, which makes it clinically valuable for the transthoracic puncture biopsy.

## Figures and Tables

**Table 1:** Baseline characteristics of patients in the two groups.

Characteristic	June 2012 to July 2013	July 2011 to May 2012	*P* value
Number of patients (*n*)	90	60	
Age (y)	59.0 ± 11.6	61.4 ± 14.1	0.273
Male/female (*n*)	54/36	41/19	0.299
Lesion location			
Pulmonary (*n*)	74	49	0.415
Mediastinum (*n*)	10	4	
Pleural (*n*)	6	7	
Malignant/benign (*n*)	66/24	36/24	0.086

Values are mean ± SD where applicable.

**Table 2:** Not suitable for biopsy after CEUS examination in CEUS group, % (*n*).

Characteristic	CEUS group
Total	8.9% (8/90)
Lung consolidation	37.5% (3/8)
Deep and smaller than 2 cm	25.0% (2/8)
Necrosis larger than 80%	37.5% (3/8)

**Table 3:** Final diagnosis of CEUS group and US group after biopsy.

Characteristics	CEUS group	US group
Malignant	**61**	**36**
Lung squamous carcinoma	23	16
Lung adenocarcinoma	25	13
Small cell carcinoma	6	3
Metastatic carcinoma	5	2
Neuroendocrine carcinoma	2	2
Benign	**21**	**24**
Inflammatory pseudotumor	6	8
Pneumonia with consolidation	7	6
Tuberculosis	3	3
Lymphoma	2	4
Thymoma	3	3

Total	82	60

**Table 4:** Accuracy of biopsy diagnosis, pulmonary atelectasis, and necrosis detection rate in CEUS group and US group, % (*n*).

Group	Size (cm)	Diagnosis accuracy				Accompany	
		Total	<3 cm	3~5 cm	>5 cm	Atelectasis	Necrosis
CEUS	6.5 ± 2.8	96.3% (79/82)	100% (10/10)	87.5% (21/24)	100% (48/48)	13.4% (11/82)	43.9% (36/82)
US	6.7 ± 3.4	80% (48/60)	100% (9/9)	77.8% (7/9)	76.2% (32/42)	1.7% (1/60)	6.7% (4/60)

*P* value	0.576	0.002	1.000	0.488	<0.001	0.013	<0.001

There was a statistically significant differences in the diagnosis accurate rate between the CEUS and US groups (*P* = 0.002) and statistically significant differences in the atelectasis and necrosis detection rate between the two groups (*P* = 0.013, *P* < 0.001). CEUS, contrast enhanced ultrasound; US, ultrasound.

**Table 5:** Post hoc analysis of a subgroup of 12 patients in the US group with a false-negative diagnosis based on initial biopsy.

Final pathologic diagnosis	No. of lesions
Necrotic tissue	4
Fibrous tissue with necrosis	2
Fibrous tissue and skeletal muscle tissue	3
Granulomatous inflammation with necrosis	1
Adipose tissue	2

Total	12

**Table 6:** Complication rate and puncture attempts in CEUS group and US group.

Group	Complication, % (*n*)	Puncture attempts
CEUS	2.4% (2/82)	2.5 ± 0.7
US	3.3% (2/60)	2.6 ± 0.6

*P* value	1.000	0.462

Values are mean ± SD where applicable. CEUS, contrast enhanced ultrasound; US, ultrasound.
